# Psychological effects of mould and damp in the home: scoping review

**DOI:** 10.1080/02673037.2023.2286360

**Published:** 2023-11-30

**Authors:** Samantha K. Brooks, Sonny S. Patel, Dale Weston, Neil Greenberg

**Affiliations:** a Department of Psychological Medicine, King’s College London, Weston Education Centre, London, SE5 9RJ, UK; b Department of Global Health and Population, Harvard T.H. Chan School of Public Health, Boston, MA, USA; Transcultural Conflict and Violence Initiative, Georgia State University, Atlanta, GA, USA; c Behavioural Science and Insights Unit (BSIU) Evaluation and Translation Directorate, UK Health Security Agency, Porton Down, Salisbury, SP4 0JG, UK

**Keywords:** Damp, Housing, Mental health, Mould, Wellbeing

## Abstract

People spend a substantial amount of time at home, so it is important that homes are safe, healthy environments. Damp and mould represent common housing problems but little is known about their potential psychological effects. This scoping review explores existing literature on the relationship between household damp/mould and psychological wellbeing. Systematic searches of six databases were conducted, supplemented by hand-searches. Thirty studies were included; 21/24 (87.5%) found significant univariate associations between damp/mould and psychological outcomes and 13/17 (76.5%) found that damp/mould remained significant independent predictors in multivariate analyses. Qualitative data from six studies revealed that participants feared potential physical health consequences of damp/mould and felt self-conscious about clothes/homes smelling damp. Our findings suggest that exposure to damp and mould accounts for a significant amount of variance in psychological outcomes. Improving housing quality, ensuring healthcare professionals are aware of the psychological health effects of damp/mould and campaigns to educate the public about how to remove damp and mould may be useful.

## Introduction

Globally, people spend much of their time at home. In the early 2000s, studies from Germany, Canada and the United States estimated that the average person spent around 65% of their time at home (Brasche & Bischof, 2005; Leech *et al*., 2002). This proportion is likely to have substantially increased since the COVID-19 outbreak in December 2019 with stay-at-home orders across the world (Mathieu *et al*., 2020) effectively forcing people to spend much of their time in their homes. Despite mobility restrictions being eased, many people are still spending much of their time at home - for example, in the United Kingdom, over 80% of workers report planning to continue working from home at least some of the time (Office for National Statistics, 2022). Therefore given the current circumstances following the COVID-19 pandemic, with individuals spending more time at home than ever before, it becomes increasingly crucial for homes to be safe and healthy environments.

The interconnection between health and the built environment (encompassing the physical structures and surroundings in which individuals live) within communities is well-established, with housing impacting not only physical wellbeing but also overall quality of life (Bonnefoy, 2007; Shaw, 2004) and shaping individuals’ behaviour, relationships, and experiences (Patel, 2022; Rashidfarokhi & Danivska, 2023). As an essential component of the built environment, housing serves as more than just a shelter, providing individuals with safety, privacy, security, and comfort. People develop emotional and meaningful relationships with their homes (Kearns *et al*., 2000; Shaw, 2004) which contribute to social resilience (Patel, 2022).

Housing is widely recognised as a social determinant of health; it has even been argued that housing is the *most* powerful determinant of health (Leifheit *et al*., 2022). There are a number of ways in which the quality and conditions of housing (or lack of housing) can substantially impact both physical and mental health (Rolfe *et al*., 2020). Emotional aspects of the home, physical housing conditions, and the social environment of the neighbourhood where the house is located all affect health and wellbeing (Novoa *et al*., 2014). For example, good housing conditions - including clean air, access to clean water, and protection from environmental hazards - are essential for preventing communicable diseases and respiratory problems, while poor housing conditions can exacerbate physical health problems (Krieger & Higgins, 2002). Insecure housing, living in unsafe neighbourhoods and unhealthy housing conditions also contribute to stress and mental health problems, and neighbourhood quality (including the quality of social relationships with others in the local area) is also an important determinant of wellbeing (Rolfe *et al*., 2020).

The impact of housing on health is particularly prominent at intersections of socioeconomic status, race, gender, sexual orientation and immigration status (Dewilde, 2022; Leifheit *et al*., 2022; Vásquez-Vera *et al*., 2022). The structural oppression of certain groups and unequal distribution of power and resources means that communities who are marginalised at any of these intersections are disproportionately affected by limited or uncertain access to adequate housing and consequently poorer health (Leifheit *et al*., 2022). For example, research suggests that the health of cisgender women, transgender individuals and non-binary individuals is more severely affected by housing problems than that of cisgender men, potentially due to the patriarchal systems operating in society which make these groups more susceptible to living in precarious housing conditions (Vásquez-Vera *et al*., 2022). It has also been suggested that Black communities experience greater barriers to housing security (Leifheit *et al*., 2022), in part due to historical injustices such as the denial of mortgages for majority-Black neighbourhoods in the United States (Leifheit *et al*., 2022) and the institutional discrimination in the United Kingdom which historically saw Black and other minority ethnic households being more likely to be offered poor quality homes (King, 2021).

There are a number of housing-related problems that people may experience, such as overcrowding, cold, damp and mould, infestation, noise from neighbours, insufficient light, and inadequate heating (Pevalin *et al*., 2008). Household damp and mould have attracted particular attention from the media in the United Kingdom since 2022 due to the ‘cost-of-living’ crisis (e.g. Anderson, 2022; Phillips, 2022), in which increased energy prices have led many households to avoid switching the heating on to keep energy bills affordable. While avoiding heating a home may reduce bills, inhabitants are subsequently less likely to open windows (in order to conserve heat), which increases the risk of mould and damp developing (Sharpe *et al*., 2015). Socioeconomic status and behaviours to mitigate the effects of fuel poverty are of course not the only causes of household damp and mould; poor construction, severe weather events such as flooding, and inadequate maintenance of the home are also causal factors (Coulburn & Miller, 2022). Concerns about climate change have also brought attention to the risks of damp and mould, as changes in temperature and humidity may result in conditions favourable for mould growth (Hayles *et al*., 2022) and climate change mitigation policies which focus on energy efficiency in indoor dwellings could reduce ventilation rates and promote mould growth (Vardoulakis *et al*., 2015).

The prevalence of damp and mould in homes varies from country to country depending on climate and housing stock. In the two years to 2019, around 3% of households in England were reported to have damp in at least one room (UK Government, 2020); until more recent statistics are published, it is unclear how much impact the cost-of-living crisis may have on the prevalence of damp, mouldy homes in the United Kingdom. In a 2007 study of eight European cities, the World Health Organization (2007) found visible mould growth in approximately 25% of the homes surveyed. Prevalence of mould in homes in the United States varies from study to study, averaging out to an estimate of around 50% of homes affected (Indoor Air Quality Scientific Findings Resource Bank: Berkeley Lab, 2023). It is also important to note the intersections of race and socioeconomic status with household damp and mould: unhealthy housing has been found to disproportionately affect ethnic and racial minorities, and those with low income (Tilburg, 2017). In the United Kingdom, whilst 3% of White British households were reported to have damp problems, this rose to 8% of Pakistani households, 9% of Black African households, 10% of Bangladeshi households and 13% of Mixed White and Black Caribbean households (UK Government, 2020). Additionally, people in rented homes – who tend to have lower incomes than those who own their homes – are significantly more likely to experience damp (Ellaway & Macintyre, 1998).

The presence of mould in the home has been associated with lower self-reported health (Adamkiewicz *et al*., 2014); neurological, skin and allergic symptoms and mucosal irritation (Tuuminen & Rinne, 2017); respiratory complaints, eye symptoms, and mucous membrane irritation (Portnoy *et al*., 2005); asthma (Moses *et al*., 2019); headaches, memory problems, nosebleeds, and body aches (Žuškin *et al*., 2009). Early-life exposure to mould has been associated with poorer cognitive function (Jedrychowski *et al*., 2011) and asthma, wheeze, allergic rhinitis and eczema (Du *et al*., 2022) in children. Household damp and mould can even be fatal: in the United Kingdom, an inquest into the tragic death of two-year-old Awaab Ishak concluded that the toddler died from a respiratory condition caused by exposure to mould in the home (BBC News, 2023). Overall, damp and mould impose a large disease burden in terms of hospitalisations and deaths – greater than other household factors such as crowding and cold housing (Riggs *et al*., 2021).

However, while the physical health effects of mould in the home have been well-documented, less is known about the psychological health effects. Housing improvement interventions (including central heating installation, reroofing, replacement windows, thermal insulation, draught-proofing and re-wiring) have been shown to have moderately strong positive effects on adult mental health (Liddell & Guiney, 2015). Receipt of fabric works in particular (i.e. structural repair which would affect damp and mould) is associated with higher likelihood of recovery from mental health problems (Curl & Kearns, 2015). It seems intuitive to hypothesise that if interventions *improving* housing problems such as damp and mould positively affect mental health, then the *presence* of damp and mould may have negative mental health effects. While many studies have examined the mental health effects of ‘housing problems’ – including damp and mould along with other problems such as noise, cold, and poor state of repair as one combined variable (e.g. Macintyre *et al*., 2003) – less is known about household damp/mould as independent predictors of mental health and wellbeing. Anecdotal reports suggest that having mould at home can cause feelings of shame (Webster, 2015) and it is possible that the sight or smell of mould in a place where people are supposed to feel particularly safe and secure might lead to feelings of anxiety, disgust, or lack of control. This review therefore aimed to explore the psychological impact of living in a home with damp and/or mould. The review was designed to be a scoping review, broad in nature to identify a wide range of literature and examine a) the extent of literature exploring direct and indirect psychological effects of damp/mould exposure and b) gaps in current research.

## Method

This scoping review followed Arksey and O’Malley’s (2005) review framework, consisting of five stages: identifying the research question, identifying relevant studies, study selection, charting the data and collating, summarising and reporting results. Within this framework we also incorporated the PRISMA guidelines for scoping reviews (Tricco *et al*., 2018).

### Identifying the research question

Given that this research was designed to be a scoping review, exploring potential wellbeing effects of household damp and mould which have not yet been systematically reviewed, our research question was broad: *What might be the psychological effects of living in a home with damp and/or mould?* To answer this question, we were interested in:

- Studies reporting on the prevalence of mental health problems, psychological problems and poor wellbeing in individuals exposed to damp/mould in the home (ideally with a comparison group of non-exposed individuals, but studies without comparison groups were also considered to be relevant)
- Studies with multivariate analyses, considering whether damp and mould were *independently* associated with psychological outcomes
- Qualitative research where participants might discuss their own perceptions of how damp/mould exposure affected their lives and wellbeing.

It is important to note here that the exposure we were interested in was the presence of either damp, visible mould, odour of mould, mildew, or condensation in the household. Outcomes we were interested in were mental health disorders (such as depression) as well as psychological outcomes such as impacts on mood, emotions, distress and wellbeing. Although it is possible that mould toxins could affect mental health through altering biochemical brain pathways (Shenassa *et al*., 2007) it is important to emphasise that in this review we were interested in only the psychological aspects of mental health.

### Identifying relevant studies

To identify studies relevant to the research question, we developed three search strategies. Search 1 included terms relating to psychological outcomes (e.g. “mental health”, wellbeing, depression) which were all combined using the Boolean operator “OR”. Search 2 included terms relating to mould or damp (e.g. mould, mold, damp) which were combined using the “OR” operator. Search 3 included terms relating to the home (e.g. housing, home) which again were combined using “OR”. The three searches were then combined using the Boolean operator “AND”. Although the British English spelling “mould” has been used throughout the manuscript we acknowledge the American English spelling “mold”, and both were accounted for in the search strategy. The full list of search terms is presented in Appendix 1.

The following databases were searched on December 2^nd^ 2022: Embase, PsycInfo, Medline, Web of Science, Global Health, and Health Management Information Consortium. Google Scholar and the preprint server medRxiv were hand-searched, assessing the relevance of (up to) the first 50 results for a number of different keyword searches (see Appendix 2). The reference lists of all included papers were also hand-searched for citations which may not have been picked up by our searches.

### Study selection

We developed the following inclusion criteria for study selection:

- Written in English (the language spoken by the authors)
- Must contain primary data (i.e., not literature reviews)
- Journal articles, letters, government reports, preprints, and conference abstracts were all acceptable formats (providing other criteria were met)
- Must explore, either quantitatively or qualitatively, the psychological effects of damp/mould exposure
- Participants needed to have been exposed to mould in the home (not in the workplace or elsewhere)
- If not all participants had experienced damp/mould, then data on mould/damp-exposed participants needed to be clearly separate from data on non-mould exposed participants
- Damp/mould needed to be a separate variable rather than combined with other housing problems
- Population of the study needed to be larger than one (i.e., no case studies)
- Damp/mould should not be the result of flooding; studies examining the psychological impact of having damp/mould in the home as a result of being flooded were excluded as it would be difficult to disentangle the psychological effects of mould and the psychological effects of experiencing a disaster.

There were no limits relating to publication year, study location, or demographic characteristics of participants.

Initial screening was carried out independently by the first author. The second author double-screened approximately 10% of citations and there was 100% agreement between the authors as to which of these citations should be included.

### Charting the data

Key data from each included paper were extracted into a Microsoft Excel spreadsheet with the following headings: authors, year of publication, country, design of study, number of participants, socio-demographic characteristics of participants, how the presence of damp/mould was assessed, wellbeing outcomes assessed, measures used to assess outcomes, conclusions, and limitations. Data were extracted from a subset of approximately 10% of papers by the second author and were found to be in agreement with the first author’s extraction.

### Collating, summarising and reporting results

First, in order to establish the frequency and nature of relationships between damp/mould and psychological outcomes, all quantitative studies containing data on the prevalence of psychological problems were grouped together. For each study, we noted whether the data indicated no psychological impact of damp/mould or whether there was significant evidence of psychological impact in univariate analysis, and additionally whether this remained significant in multivariate analysis. Given that the studies were disparate in terms of the outcomes assessed and the measures used, it was difficult to directly compare them; no meta-analysis could be performed and so the results are presented narratively, listing the studies which found significant negative psychological impact. Extracted qualitative data were synthesised using insights from thematic analysis (Braun & Clarke, 2006), with data coded and grouped together by code – for example, all data relating to negative emotions caused by damp/mould were grouped together and coded as ‘impact’, and all data relating to reasons why damp/mould caused negative emotions were grouped together and coded as ‘explanations for impact’. These are discussed narratively within the sub-section ‘qualitative findings’.

## Results

**Figure 1.**
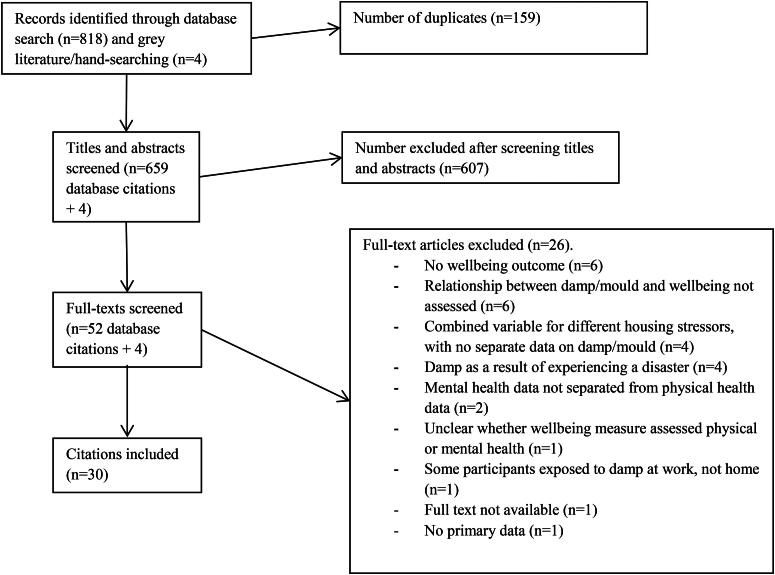
Screening process.

Database searches yielded 818 citations. These were imported into EndNote reference management software, where 159 duplicate citations were removed. Titles and abstracts of the remaining 659 citations were screened, with 531 being excluded based on title and 76 excluded based on abstract. We then searched for full texts of the remaining 52 studies. One full-text was not available and 25 were excluded after reading the full paper. This left 26 studies for inclusion in the review. One report was added after searching grey literature and three studies added after hand-searching. Overall, 30 studies were included. A flow diagram of the screening process can be seen in Figure 1.

The included studies were based in a number of countries: United Kingdom (*n* = 14), United States of America (*n* = 4), New Zealand (*n* = 3), Australia (*n* = 2), and Canada, Denmark, Finland, Germany, Guyana and Sweden (*n* = 1 each). A further two studies included multiple countries in Europe. Study populations ranged from 6 – 42,723.

Supplementary File 1 provides a summary of the characteristics of each of the included studies. Table 1 shows whether significant associations were found between mould/damp and psychological outcomes, in both univariate analyses and multivariate analyses.

**Table 1.** Univariate and multivariate associations between damp/mould and psychological outcomes.

Study	Significant univariate association between mould/damp and psychological wellbeing?	Significant association between mould/damp in the home and psychological wellbeing after adjusting for confounding variables?	Confounding variables included in model	Other housing-related variables independent predictors of psychological outcomes?*
Baird *et al*. (2022)	**✓**	**✓**	Ethnicity, sex, autism spectrum disorder, family structure, poverty, maternal depression	**High spatial density**, access to private green space, air pollution, urbanicity/rurality of the area
Blackman *et al*. (2001)	X	X	Chronic respiratory ill-health, age, serious draughts, perceived safety of the area	Household type, overcrowding, housing tenure, dwelling type, happiness with home, ability to keep warm, **draughts,** vermin, various housing defects, perception of the area, dislikes about the area, **safety of the area,** burglary in past year
Blay *et al*. (2019)	–	–	–	–
Boomsma *et al*. (2017)	**✓**	X	Affordability concerns, gender	–
Bower *et al*. (2021)	X (anxiety) **✓**(depression) **✓**(loneliness)	X (anxiety) X (depression) **✓** (loneliness)	Perceived neighbourhood-belonging, demographic characteristics (age, gender, birth country, sexuality/gender identity, relationship status, education, employment, household income, lifetime mental health diagnoses, past homelessness)	Access to outdoor space, **natural light (depression, loneliness),** internet access, **frequency of noise bothering people at home (anxiety, depression, loneliness), perceived affordability of housing (depression, loneliness),** dwelling type
Butler & Sherriff (2017)	–	–	–	–
Butler *et al*. (2003)	**✓**	**✓**	Age, ethnicity, education, marital status, birthplace, number of years lived in New Zealand, household income	**Cold home**
Casas *et al*. (2013)	**✓**	**✓**	Cohort, region, sex, parental education, child age, maternal age at birth, time spent in front of TV/computer, older siblings, asthma, environmental tobacco smoke, single mother, pet ownership	**Pet ownership**
Groot *et al*. (2022)	**✓**	–	–	–
Guite *et al*. (2006)	**✓**	X	Age, sex, requesting re-housing, housing benefit, house ownership type, number of children in the household, employment	Not liking the look of the estate/road, **neighbour noise, over-crowdedness, dissatisfaction with green spaces,** dissatisfaction with community facilities, dissatisfaction with social and entertainment facilities, **feeling unsafe to go out in the day** or night, needles and syringes left around, not enough places to stop and chat, not enough events to get people together; dissatisfaction with light, dissatisfaction with heat, dissatisfaction with draught, maintenance of grassy areas, maintenance of trees/flowers, maintenance of entrances, maintenance of play areas, age of housing, number of storeys, street noise, noise in the home, type of brickwork/frame, sport/exercise facilities, shopping facilities, insufficient street lights, vandalism, graffiti, abandoned cars, rubbish, agreement with whether people could influence decisions
Hopton & Hunt (1996)	**✓**	**✓**	Chronic illness, living in a low-income household, living with children under 16, being unemployed, moved to current dwelling because of ill health	–
Hyndman (1990)	**✓**	**✓**	Age, sex, smoking, class, dusty occupations	–
Kang *et al*. (2022)	X	–	–	–
Karunanayake *et al*. (2021)	**✓** (visible mould) X (dampness, mouldy smell)	**✓** (visible mould)	Sex, age, body mass index, trouble going to sleep or staying asleep	Dampness, mouldy smell, smoke inside house, crowding, sleeping arrangement, afraid to sleep in the home
Kilburn (2003)	**✓**	–	–	–
Kilburn (2009)	**✓**	–	–	–
Martin *et al*. (1987)	**✓**	**✓**	Number of children in the household, cigarette smoking in house	–
Midouhas *et al*. (2019)	**✓**	–	–	–
Nasim (2022)	**✓**	**✓**	Housing type, family size, number of older siblings, housing quality (damp and mould, living and bedroom temperature, overcrowding, pests, water leaks, satisfaction with home); neighbourhood quality (social networks, extent to which crime affects their life) – individual statistics not presented, but decomposition analysis of mediators suggested 40% of the indirect effect *via* housing quality was captured by damp and mould	–
Oudin *et al*. (2016)	X	–	–	–
Packer *et al*. (1994)	**✓**	X	Age, sex, social class	–
Paterson *et al*. (2018)	**✓**	X	Ethnicity, marital status, education, employment	House too small, hard to get to from the street, **in poor condition**, too cold or difficult to heat, **presence of pests such as mice or insects,** too expensive, any other unspecified housing problem
Platt *et al*. (1989)	**✓** (‘bad nerves’) X (depression)	**✓** (‘bad nerves’) X (depression)	Economic position, cigarette smoking, length of time at the address, other housing problems (or cold alone), household income	–
Seppala *et al*. (2022)	–	–	–	–
Serjeant *et al*. (2022)	–	–	–	–
Shenassa *et al*. (2007)	**✓**	**✓**	Perception of control over one’s home, physical health	**Crowding,** ventilation, natural light, heating, perception of control, **exposure to cigarette smoke**
Shortt & Rugkåsa (2007)	–	–	–	–
Wen & Balluz (2011)	**✓**	**✓**	Age, sex, education level, employment, state, chronic disease	–
World Health Organization (2007)	**✓**	**✓**	Age, gender, length of residency, socioeconomic status, tobacco smoke exposure	**Number and quality of green areas, dust, satisfaction with light in the home,** building age, urbanisation, traffic noise, feeling insecure in the area at night
Ziersch *et al*. (2017)	–	–	–	–

* Significant independent predictors of psychological outcomes are presented in **bold**.

### Univariate associations between household mould/damp and psychological wellbeing

#### Significant findings

Twenty-four of the included studies carried out statistical tests assessing the relationship between damp/mould and psychological outcomes and 21 of these (87.5%) found significant associations. Univariate analyses found significant associations between mould/damp in the home and stress (Packer *et al*., 1994); psychological distress (Hopton & Hunt, 1996; Paterson *et al*., 2018); anxiety (Packer *et al*., 1994); depression (Bower *et al*., 2021; Hyndman, 1990; Packer *et al*., 1994; Shenassa *et al*., 2007; World Health Organization, 2007); postnatal depression (Butler *et al*., 2003); affective disorders (Groot *et al*., 2022); dissatisfaction with life (Boomsma *et al*., 2017); loneliness (Bower *et al*., 2021); low vitality (Guite *et al*., 2006); reduced vigour (Kilburn, 2003); poor sleep (Karunanayake *et al*., 2021); ‘nerves’ (Platt *et al*., 1989); poor wellbeing (Boomsma *et al*., 2017; World Health Organization, 2007); and poor general mental health (Guite *et al*., 2006; Kilburn, 2003; Wen & Balluz, 2011).

Univariate analyses focusing on children found significant associations between household mould/damp and conduct problems (Baird *et al*., 2022; Midouhas *et al*., 2019); emotional symptoms (Casas *et al*., 2013; Martin *et al*., 1987; Midouhas *et al*., 2019); ‘nerves’ (Martin *et al*., 1987); irritability (Platt *et al*., 1989); feeling depressed/unhappy (Platt *et al*., 1989); mental health (Nasim, 2022); and ‘total difficulties’ (combination of emotional symptoms, conduct problems, hyperactivity/inattention and peer relationship problems) (Casas *et al*., 2013).

#### Non-significant findings

In a minority of studies (*n* = 3), univariate analyses revealed no significant associations between household mould/damp and any of the psychological outcomes examined, including psychological distress (Blackman *et al*., 2001); stress (Kang *et al*., 2022; Oudin *et al*., 2016); fatigue (Oudin *et al*., 2016); concentration difficulties (Oudin *et al*., 2016); sleep problems (Oudin *et al*., 2016); and overall mental health (Kang *et al*., 2022).

A further three studies which found univariate associations between household damp/mould and *some* psychological outcomes also found non-significant results for other outcomes. A study which had found a significant association between damp/mould and ‘nerves’ (Platt *et al*., 1989) found no significant association between damp/mould and feeling depressed/down-hearted. Bower *et al*. (2021) found no significant association between damp/mould and anxiety, although they did find that damp/mould were significantly associated with both depression and loneliness. Additionally, Casas *et al*. (2013) found no significant relationship between household damp/mould and conduct problems, hyperactivity/inattention or peer relationship problems, although they did find significant relationships with emotional symptoms and total difficulties.

### Multivariate associations between household mould/damp and psychological wellbeing

#### Significant findings

In 13/17 (76.5%) studies, associations between household mould/damp and psychological outcomes remained significant after adjusting for confounding variables (see Table 1). Mould and damp remained associated with anxiety (World Health Organization, 2007); depression (Hyndman, 1990; Shenassa *et al*., 2007; World Health Organization, 2007); postnatal depression (Butler *et al*., 2003); loneliness (Bower *et al*., 2021); sleep problems (Karunanayake *et al*., 2021); ‘bad nerves’ (Platt *et al*., 1989); overall mental health or wellbeing (Wen & Balluz, 2011; World Health Organization, 2007); psychological distress (Hopton & Hunt, 1996); and children’s conduct problems (Baird *et al*., 2022), emotional problems (Casas *et al*., 2013), mental health (Nasim, 2022) and ‘bad nerves’ (Martin *et al*., 1987). Covariates included in the multivariate regressions can be seen in Table 1.

Shenassa *et al*. (2007) found that perception of control over one’s home attenuated the association between mould and depression, and inclusion of physical health problems in the regression model (particularly chronic respiratory problems) also attenuated the association. However, these two mediators still did not account for the entire association between mould and depression, which remained significant.

#### Loss of significance

In six studies which found significant associations between household mould/damp and psychological outcomes, significance of at least one of these relationships was lost after adjusting for confounding variables. These studies showed a loss of association between mould/damp and depression (Bower *et al*., 2021; Packer *et al*., 1994); anxiety/stress (Packer *et al*., 1994); distress (Paterson *et al*., 2018); overall wellbeing (Boomsma *et al*., 2017); general mental health (Guite *et al*., 2006); and irritability and feelings of depression/unhappiness in children (Platt *et al*., 1989).

Confounding variables included in regression models which no longer showed mould/damp as significant predictors included demographics such as age, gender, ethnicity, and social class (Bower *et al*., 2021; Guite *et al*., 2006; Packer *et al*., 1994; Paterson *et al*., 2018); marital status (Paterson *et al*., 2018); education (Paterson *et al*., 2018); employment status (Paterson *et al*., 2018); having nobody in the household employed (Platt *et al*., 1989); overcrowding (Platt *et al*., 1989); housing affordability concerns (Boomsma *et al*., 2017); perceived neighbourhood belonging (Bower *et al*., 2021); and living with smokers (Platt *et al*., 1989).

In these studies, housing factors which did emerge as predictors of psychological outcomes in multivariate analyses included poor condition of the home (Paterson *et al*., 2018) and presence of pests in the home (Paterson *et al*., 2018).

One study (Karunanayake *et al*., 2021) found that while exposure to visible mould remained a significant predictor of poor sleep after adjusting for confounding variables, dampness alone and a mouldy smell alone lost significance.

#### Other quantitative findings

One study compared mental health outcomes in mould-exposed populations compared to populations with other conditions: Kilburn (2009) found that mood states (a variable consisting of the sum of five adverse moods) for a mould exposure group were slightly lower than for a chemical exposure group, but nearly twice the group exposed to neither.

One study did not statistically assess the relationship between damp/mould exposure and psychological outcomes, but instead asked participants whether they believed that exposure had affected the mental health of people in their household. Shortt & Rugkåsa (2007) asked participants their perceptions on whether condensation, damp or mould had any impact on the mental health of people in their household and found that very few respondents felt that they had any impact. It is unclear from this study whether this was indeed the case or whether there were, in fact, any impacts and these were simply not recognised.

#### Qualitative findings

Six studies included qualitative data relating to participants’ experiences of damp/mould in their homes, all of which focused on tenants and those in social housing provided by housing associations or councils, rather than owner-occupiers. Participants described a number of negative wellbeing effects of having household damp/mould, including fear of the potential health hazards (Blay *et al*., 2019; Boomsma *et al*., 2017; Butler & Sherriff, 2017); unhappiness (Blay *et al*., 2019; Ziersch *et al*., 2017); depressive symptoms (Ziersch *et al*., 2017); anger (Blay *et al*., 2019; Ziersch *et al*., 2017); distress (Butler & Sherriff, 2017); discomfort (Blay *et al*., 2019); frustration (Boomsma *et al*., 2017; Butler & Sherriff, 2017); and feeling ‘on edge’ (Blay *et al*., 2019), all of which participants attributed to the sight, smell and knowledge of health implications of mould and damp. Sadness, anger and depressive symptoms appeared to be particularly pronounced for asylum seekers who felt a greater lack of control than those with permanent resident status over housing issues (Ziersch *et al*., 2017).

Damp and mould were described as ‘a nuisance’ (Blay *et al*., 2019) and ‘depressing’ (Boomsma *et al*., 2017), with participants fed up with having to frequently paint over mould and damp (Boomsma *et al*., 2017), living with an unpleasant smell (Butler & Sherriff, 2017; Serjeant *et al*., 2022) and having clothes ruined by mildew and mould (Serjeant *et al*., 2022). Participants reported feeling self-conscious that the smell of mould would linger and others would smell damp on their clothes when out in public or welcoming visitors to their homes (Butler & Sherriff, 2017); the smell was seen as not only a threat to home comfort but to participants’ identities as capable and independent adults. Participants in three studies reported having had their physical health or that of their families negatively affected by damp and mould (Boomsma *et al*., 2017; Butler & Sherriff, 2017; Ziersch *et al*., 2017). Some reported increased arguments with other occupants in their homes due to others’ lack of understanding of the negative impact of dampness and tensions around the increase in energy bills as a result of opening windows and using heating (Blay *et al*., 2019).

Participants in all qualitative studies wanted to reduce damp and mould in their homes, wanting a more ‘healthy’ living environment (Blay *et al*., 2019), and although most understood how they could reduce damp and mould (e.g. through aeration or dehumidifiers) many avoided or limited the duration of these behaviours due to consciousness of energy bills and fear of cold (Blay *et al*., 2019; Serjeant *et al*., 2022).

Tenant-landlord relationships could be strained, which meant that attempts to resolve issues relating to damp and mould tended to be unsuccessful (Butler & Sherriff, 2017); participants with landlords responsive to damp issues reported feeling lucky (Serjeant *et al*., 2022). Others were less lucky, describing how landlords made unrealistic and unhelpful suggestions such as keeping the heating on low all the time, which they could not afford to do (Butler & Sherriff, 2017).

Finally, Seppala *et al*. (2022)’s participants described how their experiences with poor indoor air quality were ‘delegitimised’, reporting how doctors would not testify that their illnesses were caused by mould and that authorities claimed that there was nothing wrong with their apartments despite evidence of mould, so they could not be given new accommodation. These experiences were perceived to be unfair and associated with anger and bitterness.

## Discussion

This scoping review of 30 studies found that the majority of quantitative studies (87.5%) showed a univariate association between household damp/mould and a range of psychological outcomes, including stress, depression, anxiety, and poor overall mental health and wellbeing. Of the studies which carried out multivariate analyses, 76.5% found that household damp/mould remained a significant independent predictor of mental health outcomes even when controlling for multiple other variables including socioeconomic status, house ownership status, employment and chronic illness. Additionally, qualitative data from six studies revealed that participants believed household damp and mould affected their wellbeing, citing negative feelings elicited by the sight and smell of mould; fear of potential physical health consequences; and embarrassment about clothes and homes smelling damp. While the relationships between household damp/mould and mental health and wellbeing are not universal, and there are caveats – which we discuss below – this review provides the first evidence of a substantial association between damp/mould and mental health/wellbeing.

People exposed to damp/mould may be more prone to physical health issues, which can further impact mental health: the link between physical health and mental health is well-established (Ohrnberger *et al*., 2017). Also, it may be the physical health effects – or fear of physical health effects – associated with damp and mould which impact on psychological wellbeing. Indeed, the qualitative data we reviewed found that fear of physical health consequences was a prominent theme in 3/6 studies. It would be worthwhile to investigate the extent to which this fear may be fuelled by media coverage. Some participants in the qualitative data felt that healthcare staff had not supported the idea that their physical health had been damaged; while we cannot ascertain the extent to which damp and mould may truly have affected physical health, it is possible that fear-inducing messages in the media may influence participants (Maran & Begotti, 2021).

We also highlight Shenassa *et al*.’s (2007) important finding that perception of control over one’s home attenuated the association between mould and depression. Notably, in this study approximately one-quarter of participants rented properties and the rest owned their homes. It is likely that renting is associated with perceived lack of control due to the precarity of living in rented accommodation, which has been exacerbated after the COVID-19 pandemic (Newton *et al*., 2022; Waldron, 2023). Only one other study in this review (Guite *et al*., 2006) included home ownership status in a regression model, finding that the relationship between damp/mould and psychological wellbeing lost significance in a multivariate model which included home ownership status.

Despite the finding that household damp/mould remained an independent predictor of mental health in 76.5% of studies with multivariate analyses, it remains difficult to fully disentangle the effects of damp and mould. Many of the studies reviewed are cross-sectional, making it difficult to clearly understand the directionality of relationships – we can only note associations, rather than causation. Researchers have urged caution when interpreting findings showing a link between mould and depression (Potera, 2007), suggesting it is not possible to infer a causal relationship between the two and proposing that income may be an important confounding variable. Those with damp/mould might also be more likely to have difficulty paying fuel bills – financial difficulties are associated with poor mental health (Knifton & Inglis, 2020) and it is difficult to disentangle how much wellbeing is affected specifically by damp/mould in the home and how much it is affected by the myriad other problems that might be caused by poverty. Our review found that the relationship between damp/mould and psychological wellbeing persisted after adjusting for socioeconomic status in four studies (Baird *et al*., 2022; Hopton & Hunt, 1996; Platt *et al*., 1989; WHO, 2007) whereas the relationship lost significance after adjusting for socioeconomic status in three studies (Boomsma *et al*., 2017; Bower *et al*., 2021; Packer *et al*., 1994). There could be other confounders such as education: higher education equips individuals with good communication skills (Iksan *et al*., 2012), therefore individuals with higher levels of education might find it easier to address problems of damp/mould through legal means or discussions with housing associations or landlords. Our review provided mixed findings on this: the relationship between damp/mould and psychological wellbeing persisted after adjusting for education in two studies (Butler *et al*., 2003; Wen & Balluz, 2011) and after adjusting for parental education in one study of children (Casas *et al*., 2013). However, in two studies (Bower *et al*., 2021; Paterson *et al*., 2018) the relationship lost significance after including education in the model. Within the literature, insufficient attention has been given to the conditions underlying inequities in the distribution of toxic exposures in the home; toxic exposures do not occur in isolation and the unfavourable social conditions associated with damp and mould are also likely to generate other environmental hazards which accumulate over time (Rauh *et al*., 2008). However, our review found that in a number of studies (Baird *et al*., 2022; Butler *et al*., 2003; Shenassa *et al*., 2007; WHO, 2007), the relationship between damp/mould and psychological wellbeing persisted after including other housing quality issues (including high spatial density, cold, crowding, access to green areas, dust, and lighting) in regression models. It also cannot be discounted that mould and other toxins may impact mental health directly through potential effects of toxic exposure on brain chemistry.

Given the methodological limitations of the reported papers, and some inconsistencies in their findings, it is not possible to draw a firm conclusion concerning the link between household damp/mould and mental health/wellbeing based on these findings. Although the lack of prospective research and potential confounding variables make it difficult to establish causation, this review is a useful first step in ascertaining whether damp/mould might play a role in psychological wellbeing, and the studies reviewed suggest that this is indeed the case. The effect persisting in many studies even when covariates are controlled for strengthens the argument for the role of damp/mould in wellbeing.

Overall, while this review could not provide clear evidence of a causal relationship between damp/mould and psychological wellbeing – because the reliance on cross-sectional research does not allow for this – we did find relatively consistent evidence suggesting an association between damp/mould and psychological health. This suggests that the topic is worthy of further investigation and that whatever the cause, it appears that people living in mouldy homes are at increased risk of reporting mental health problems. More carefully constructed studies are needed to be able to properly answer the question of how much direct impact exposure to household damp and mould may have on psychological wellbeing.

The topic is of clear public health importance and may be a proxy for poor housing issues as well as important in itself. If indeed mental health is negatively affected by poor housing such as damp and mould, it may be the case that the costs of treating poor mental health (as well as loss of tax revenue from people with poor mental health who are unable to work, and potentially intergenerational costs from children suffering the effects of poor housing) could be offset by investing in better housing options in the built environment. This review also highlights the importance of making healthcare professionals aware of damp and mould as potential risk factors for poor psychological wellbeing. Finally, our findings also contribute to the broader argument for improving standards of living in general. Instead of taking a laissez-faire approach to mould in homes, treatment of damp and mould should be prioritised as treatment is a relatively low-cost intervention and could improve wellbeing and mental health if implemented. Better guidance or support on removing household mould could be a relatively quick and easy intervention to implement – for example, national campaigns offering practical advice on reducing damp, condensation and mould as well as health information (Boomsma *et al*., 2017).

It is important that housing is recognised as a social determinant of health and that housing disparities are considered in future research. We suggest that an intersectional perspective is essential for understanding how oppression or privilege of certain groups can impact on housing and health (Vásquez-Vera *et al*., 2022). Research focusing on marginalised populations, health researchers joining housing justice movements to prioritise intersectionality, and involving people with lived experience of housing disparities in the design of research studies would all help to ensure an intersectional perspective is achieved (Leifheit *et al*., 2022).

Limitations of the current review include the decision to limit the review only to articles published in English and the fact that no systematic appraisal of the quality of included studies was performed. While quality appraisal is not a compulsory aspect of a scoping review, we acknowledge that using a standardised quality appraisal or risk of bias tool to evaluate each study would have been beneficial in order to assess the trustworthiness and value of the included studies. We also acknowledge that, while there was agreement regarding the data extraction of the 10% of studies the second author independently carried out, our review would have been more robust had the second author independently extracted data from all studies. Nevertheless, the findings offer valuable evidence of a potential relationship between mental health/psychological wellbeing and household damp and mould, linking to growing interest in the relationships between social (psychological) resilience, the built environment and urban health. This evidence provides a foundation for future research to build upon.

## Conclusion

In conclusion, this scoping review reveals consistent evidence of an association between household dampness/mould and various psychological outcomes. The majority of quantitative studies support this link, with more than three quarters of the studies showing a significant association. However, determining the direct impact of dampness/mould on psychological wellbeing is challenging due to confounding factors and the inability to establish causality. Considering the broader context of social resilience, urban health, and the built environment literature, the role of mould in understanding mental health and wellbeing should not be overlooked. The potential accumulation of environmental hazards in homes with dampness and mould, in addition to the interplay with other housing quality issues, further complicates the assessment of the specific impact of dampness/mould alone. Furthermore, the fear of physical health consequences associated with mould, and media coverage on this topic, may influence individuals’ perceptions and mental health outcomes. Further research is needed to disentangle the specific effects of dampness/mould from other interconnected factors and to inform interventions and policies aimed at improving housing standards and addressing potential public health implications. In particular, more prospective/longitudinal research is needed to explicitly try to establish a causal relationship between damp/mould and wellbeing. Despite limitations such as language restrictions and the absence of systematic quality appraisal, this review provides a valuable foundation for future research. It underscores the significance of considering household dampness/mould in the context of social resilience, urban health, and the built environment. This topic warrants further investigation to better understand the relationship, develop effective interventions, and inform housing and public health policies.

## Supplementary Material

Supplemental Material

Supplemental Material
