# Supplementary material for: Psychological effects of mould and damp in the home: scoping review
Source: Hous Stud. 2023 Nov 30;40(2):323–45. doi: 10.1080/02673037.2023.2286360 (PMC11771470; doi:10.1080/02673037.2023.2286360)
Supplement: Supplemental Material [file CHOS_A_2286360_SM4327.pdf]

Appendix 1. Search strategy for databases

1. “mental health”

2. wellbeing

3. well-being

4. depression

5. anxiety

6. trauma*

7. distress*

8. ptsd

9. resilien*

10. mood*

11. emotion*

12. coping

13. psychological

14. psychiatric

15. 1 or 2 or 3 or 4 or 5 or 6 or 7 or 8 or 9 or 10 or 11 or 12 or 13 or 14

16. mould

17. mouldy

18. damp

19. dampness

20. mold

21. moldy

22. condensation

23. mildew

24. 16 or 17 or 18 or 19 or 20 or 21 or 22 or 23

25. house*

26. housing

27. home*

28. room*

29. abode*

30. dwelling*

31. living condition*

32. living situation*

33. 25 or 26 or 27 or 28 or 29 or 30 or 31 or 32

34. 15 and 24 and 33

Note: The asterisk symbol indicates truncation, i.e. ‘distress*’’ would capture not only the term ‘distress’ but also ‘distressed’ and ‘distressing’.
