# Supplementary material for: Psychological effects of mould and damp in the home: scoping review
Source: Hous Stud. 2023 Nov 30;40(2):323–45. doi: 10.1080/02673037.2023.2286360 (PMC11771470; doi:10.1080/02673037.2023.2286360)
Supplement: Supplemental Material [file CHOS_A_2286360_SM4319.pdf]

Appendix 2: Searches on Google Scholar and medRxiv

1. mould and mental health

2. mold and mental health

3. mould and psychological

4. mold and psychological

5. mould and wellbeing

6. mold and wellbeing
